# Immunogenicity of Monoclonal Antibodies and the Potential Use of HLA Haplotypes to Predict Vulnerable Patients

**DOI:** 10.3389/fimmu.2022.885672

**Published:** 2022-06-17

**Authors:** Romy Mosch, Henk-Jan Guchelaar

**Affiliations:** Department of Clinical Pharmacy & Toxicology, Leiden University Medical Center, Leiden, Netherlands

**Keywords:** monoclonal antibodies, antidrug antibodies, human leukocyte antigen, HLA haplotypes, ntADA, biomarker

## Abstract

The use of monoclonal antibodies (mAbs) in the clinic has successfully expanded to treatment of cancer, viral infections, inflammations, and other indications. However, some of the classes of mAbs that are used in the clinic show the formation of anti-drug antibodies (ADAs) leading to loss of efficacy. This review describes ADA formation for the various mAbs, and its clinical effect. Lastly, this review considers the use of HLA-haplotypes as biomarkers to predict vulnerability of patients sensitive to formation of ADAs.

## Introduction – What Exactly Is a Monoclonal Antibody

In order to recognize and neutralize alien organisms or antigens, B-cells secrete antibodies (Abs). These Abs are glycoproteins that belong to the superfamily of immunoglobulins. The Abs consist of two heavy chains and two light chains that characterize the isotype of the Ab (1). In the clinic, therapeutic monoclonal antibodies (mAbs) usually are of the γ-immunoglobulin (IgG) isotype. For this IgG isotype, the hypervariable regions of the heavy chains and light chains connect to form the antigen binding site (Fab-domain). The two constant domains together function as the fragment crystallizable (Fc-domain), which is responsible for the effector function of the immunoglobulin. Due to these Fab and Fc-domains, the IgG molecule is bivalent (2). In mice, the neonatal Fc receptor (FcRn) regulates the serum half-life of the IgG molecule through pH-dependent antibody recycling (2, 3).

Most therapeutic mAbs can target multiple disease targets (4). Of the mAbs currently on the market, 54% are fully human, meaning that they only feature human genetic sequences. 32% of the market is humanized, and 14% is chimeric (5).

## Repeated Administration of mAbs Elicits Efficacy Reducing Immune Response

Even though mAbs are currently used in the treatment of a variety of diseases and have a promising future, their use has shown to be highly immunogenic and can cause an anti-drug antibody (ADA) response (2). These ADA responses intervene with the efficacy of a drug, or neutralizes a drug completely. Thereby, ADAs can alter the pharmacokinetic (PK) and pharmacodynamic (PD) properties of the mAb. ADAs can also eventually lead to severe adverse immune reactions in humans (5, 6).

The formation of these ADA responses is believed to depend on the interaction between the drug itself (e.g., its glycosylation, impurities, aggregation, or non-human sequences), the patient (e.g., the type of disease, genetic factors, concomitant immunomodulators) and to the route and regularity of administration of the mAb. Because the molecular mechanism of the development of these ADA responses are not fully understood, several researchers believed that the murine origin of the mAbs was the cause of the development of the immunogenic reaction. For that reason, humanized and chimeric mAbs were developed aimed at avoiding the human antimouse antibody response (HAMA) in the clinic (5, 7–9).

However, this did not eradicate the immunogenicity potential of mAbs and the related ADA response (2). Besides the questions of how and why ADA responses occur, the difficulty of the situation is further increased by the observation that some patients do develop ADA, but others don’t. It is also complicated more by the notion that the immunogenicity differences in patients receiving the same mAbs as treatment (10). These differences not only caused by regular interindividual factors, but also by geographical and racial differences (11).

The consequences of the immunogenicity elicited by mAbs range from absence of effect of the mAb to severe and life-threatening responses (12–14). Registered consequences include infusion reaction, anaphylaxis, immune complex-mediated diseases and loss of efficacy (15–21).

The anti-drug antibody responses that are elicited in mAbs-receiving patients can be classified into two categories, neutralizing ADA (*nt*ADA) which influence the binding capacity of the drug directly by targeting the antigen-binding site, and the non-*nt*ADA which recognizes other epitopes that are present on the drug while still facilitating mAb binding activity. However, the non-*nt*ADA’s can in fact be malignant (22, 23).

## ADA-Responses Elicited by mAbs and Their Clinical Effects

Over 90% of the therapeutic proteins cause immunogenicity, with the production of ADAs as a result (24). However, this immunogenic reaction is not the same in all patients and also not the same for all mAbs. The concentration of ADA can be measured (i.e., in blood), which is indicated as the titer. However, there is currently a lack of consitent and reliable immunogenicity assays to use for clinical desiscion making (25).

Some patients show a low ADA titer, in whom the concentration of free drug may still be high enough to be effective. Other patients may show a high ADA titer, causing the majority of the drug to be neutralized resulting in an absence of clinical response (23, 24, 26). As such, the formation of ADAs upon mAb use is of major clinical importance.

As the ADA responses are not only different between patients but also between mAb-classes, these were studied separately to describe which class of mAb shows an ADA response, when in the treatment that response is occurring and what the clinical effect of this response is (11). A summary of this literature study can be found in **Table 1**.

**Table 1:** Summarized overview of mAbs with corresponding type and whether ntADAs are formed or not.

Type	Name	*nt*ADA formation
Anti-TNFα	Etanercept	Non-*nt*ADA in 20 to 25% of patients (27–30).
	Infliximab	*nt*ADA in adults: 10% of patients, in children 12 to 52% of patients. Non-*nt*ADA in up to 51% of the patients (30–37).
	Adalimumab	*nt*ADA in 12-35% of patients (16, 32, 38–43).
Anti-VEGF	Bevacizumab, ranibizumab, ramucirumab	No ADA development (32–41).
Anti-CD3	Muromonab	*nt*ADA in 100% of patients (44–48).
	Foralumab	No ADA development (48–51).
Anti-CD20	Rituximab	*nt*ADA in 11 to 26.4% of patients (52–55).
	Ofatumumab	No ADA development (56).
	Obinutuzumab	Non-*nt*ADA in 7% of patients (57).
	Ocrelizumab	Non-*nt*ADA in 1 to 4.7% of patients (52, 58).
Anti-CD38	Daratumumab	Non-*nt*ADA in 1.3% of patients (59, 60).
Anti-CD52	Alemtuzumab	Non-*nt*ADA in 85% of patients (60, 61).
Anti-HER2	Trastuzumab	Non-*nt*ADA in 1% of patients (62–64).
	Pertuzumab	Non-*nt*ADA in 30% of patients (65).
Anti-EGFR	Cetuximab	Non-*nt*ADA in 5% of patients (66–69).
	Panitumumab	Non-*nt*ADA in 1.8% of patients (70–72).
	Nimotuzumab	No ADA development (73).
	Necitumumab	*nt*ADA in 1.4% of patients (74).
Immune checkpoint inhibitor-inhibiting	Ipilimumab	*nt*ADA in 26% of patients (75).
	Pembrolizumab	Non-*nt*ADA in 3.7% of patients (76).
	Nivolumab	Non-*nt*ADA in 12.7% of patients (73, 77, 78).
	Atezolizumab	Non-*nt*ADA in 13 to 54% of patients (79).
	Avelumab	Non-*nt*ADA in 14 to 19% of patients (80, 81).
Anti-IL	Tocilizumab	Non-*nt*ADA in <2% of patients (82).
	Ustekinumab	*nt*ADA in 5% of patients (83, 84).
	Secukinumab	Non-*nt*ADA in <1% of patients (85).
	Ixekizumab	Non-*nt*ADA in 17% of patients (86, 87).
Anti-IgE	Omalizumab	No ADA development (88–92).
Anti-VLA-4	Natalizumab	*nt*ADA in 4-14% of patients (93–95).

## Method

For this literature study, a search of English publications listed in the electronic databases of the NCBI through PubMed was performed. Included in this search were the terms ‘Pharmacogenomic variants’, ‘Antidrug Antibody’, ‘Human Leukocyte Antigens’ and ‘Monoclonal Antibodies’ and MESH-terms related to them. After this PubMed search, an analytical review based on inclusion of humans in ADA response development tests was performed.

## Overview of Antidrug Antibodies Elicited Against Monoclonal Antibodies and Their Clinical Effects

### Tumour Necrosis Factor Alpha Antagonistic mAbs

Tumour necrosis factor alpha (TNFα) antagonistic mAbs are used in the clinic to treat rheumatoid arthritis, spondylarthritis, psoriasis and inflammatory bowel disease. Three biologic therapies are currently approved: etanercept, infliximab and adalimumab. Etanercept is a dimeric fusion protein that blocks the effect of TNFα, infliximab is a chimeric monoclonal receptor antibody and adalimumab is a human monoclonal TNFα antibody. While all these mAbs elicit an ADA response, this response can be attenuated by immunosuppressors (especially methotrexate) as much as a reduction of 80% (16, 27). For this reduction, the mechanism is still unknown (27).

### Immunogenicity of Etanercept

ADAs against etanercept are formed in 20 to 25% of the patients receiving the drug. However, the antibodies formed are all non-neutralizing. Patients that were monitored for the formation of these antidrug antibodies in a study that lasted 120 weeks, showed an increase of antibodies with an increase in the duration of the study. Despite the presence of these antibodies, these have no correlation to the clinical response or adverse effects (16, 96). This suggests the presence of drug binding antibodies rather than drug neutralizing antibodies (27–30).

### Immunogenicity of Infliximab

In adults receiving treatment with infliximab, about 10% show development of *nt*ADAs, and this rate goes up to 51% for non-*nt*ADAs (30, 31). The incidence rates of *nt*ADAs is even higher in patients with Crohn’s Disease that receive infliximab after drug-free intervals of maximum 16 weeks (32). The patients in which these antibodies are present, infusion reactions, low titers and loss of drug efficacy are seen more often than in antibody-negative patients receiving the same treatment (16, 33–37). The development of these ADA responses is seen less often in patients that simultaneously receive immunosuppressive therapies like methotrexate (32).

In children, the incidence of ADA responses is higher. In a study that include paediatric patients with Crohn’s Disease, 12% of the tested population was antibody-positive despite the simultaneous administration of immunosuppressive therapies. In the study covering paediatric patients with ulcerative colitis, 52% of the tested population was antibody-positive after a median 24-month period. Here, there was no simultaneous administration of immunosuppressive therapies (30, 32).

### Immunogenicity of Adalimumab

In rheumatoid arthritis-patients receiving treatment with adalimumab, the patients treated with only adalimumab showed higher incidence of immunogenicity against the mAb after 6 months than patients treated with adalimumab and concomitant immunosuppressants (12% versus 1%) (32, 38–43). The levels of non-ntADAs in patients ranges up to 35% (42). No apparent correlation was shown between the development of the antibodies to the adverse reactions. The antibodies that were formed in the patients, were neutralizing. The formation of the antibodies was seen more often in patients that received their dosage every other week than it was in patients receiving their dosage weekly. The same effects were seen in patients with other diseases as is described in the Humira (adalimumab) label distributed by the FDA (16, 32).

For the anti-TNFα therapies discussed here, reports show that dose titration reduces the incidence of ADA responses and improves the drug response (18, 97). However, other studies show that this dose escalation boosts the immune response with consequences like infusion-related adverse events or severe thromboembolic phenomena (27, 98, 99). The mechanisms behind this remains unclear (27).

### Vascular Endothelial Growth Factor Antagonistic mAbs

As vascular endothelial growth factor (VEGF) is a mediator of angiogenesis related to tumours and conditions like diabetic retinopathy and age-related macular degeneration, anti-VEGF therapy is used in the clinic predominantly for therapy of solid tumours (100, 101). Anti-VEGF therapies that have been developed and are currently used in the clinic are bevacizumab, ranibizumab and ramucirumab (102). Bevacizumab is a recombinant humanized IgG mAb targeting VEGF-A, thus preventing the complex of VEGF-A and VEGFR-2 to form (102, 103). Ranibizumab is an affinity-matured antibody Fab-domain, which is developed against all fragments of the VEGF (104, 105). Ramucirumab is a fully human mAbs that inhibits VEGFR2 *via* the blockage of the VEGFR2-VEGF ligand interaction (102, 106, 107). None of the mentioned VEGF-inhibiting mAbs show immunogenicity (102–110).

### Cell Surface Antigen-Targeted mAbs

#### CD3-inhibiting mAbs

##### Immunogenicity of Muromonab

The first murine mAb available on the market was Ortho Kung T3 (OKT3, muromonab) (44). This mAb is from murine origin, and showed extreme immunogenicity with a high incidence of ADA responses, but also a wide variety of severe side effects including cytokine syndrome, seizures, encephalopathy and graft thrombosis (45–47). OKT3 is a mouse IgG2a-antibody that targets the CD3_ε_-chain in the CD3/TCR that complex characterizes T-lymphocytes (44).

Muromonab was later humanized by grafting the complementary-determining region into a structure with a human IgG-backbone, which in phase I studies did not show immunogenicity when administered orally (48).

##### Immunogenicity of Foralumab

Foralumab is a completely human anti-CD3 mAb. For this mAb, the Fc-fragment of human IgG1 was mutated so the mAb shows no FcR-binding *in vitro* and displays minor cytokine release *in vivo* while sustaining the modulation of the CD3/TCR-complex and T-cell depletion (49).

The present generation of anti-CD3 mAb presents a reduced affinity for Fc-receptors, resulting in decreased side effects when compared to the original FcR-biding antibodies that were derived from rodents (44). This generation shows very promising results in preclinical studies looking at the possibility of oral or nasal administration (48–51).

#### CD20-inhibiting mAbs

Anti-CD20 mAbs are used in the clinic to achieve B-cell depletion. Initially, this class of mAbs was developed to treat B-cell proliferative disorders such as non-Hodgkin’s lymphoma and chronic lymphocytic leukaemia, but the therapies are currently also applied in the therapy of rheumatoid arthritis and other autoimmune diseases with both T-cell and B-cell etiology (52).

##### Immunogenicity of Rituximab

Rituximab is a murine-human chimeric anti-CD20 mAb. The development of *nt*ADA responses in the clinic is seen often, which is believed to be caused by the chimeric nature of the antibody (52). A study focused on the safety of rituximab in patients with rheumatoid arthritis that administered rituximab together with methotrexate showed that 11% of the patients enrolled in the study showed an ADA response within 8 to 12 weeks after administration (53). Another study, the HERMES trial, presented 26.4% of the patients with immunogenicity against the drug at week 48 (54). Similar studies show that anti-rituximab antibodies reduce the efficacy of the drug (55).

##### Immunogenicity of New-Generation Anti-CD20 mAbs

Ofatumumab is one of the new-generation anti-CD20 mAbs currently used in the clinic (52). In one of the phase II trials that was conducted for ofatumumab, the none of the patients developed an ADA response in week 48 (56).

Obinutuzumab is another new-generation anti-CD20 mAb that is FDA-approved for the treatment of chronic lymphocytic leukaemia (52). In the clinical trials, 7% of the patients have developed an ADA response against this drug during treatment; the ADA were found in follow-up tests performed 6-months or more after treatment and did not have any effect on the efficacy of the drug. Other studies did not show development of ADA responses at all (57).

Ocrelizumab has been FDA-approved in the treatment of relapsing-remitting MS (52). Several trials were conducted to assess the safety of ocrelizumab, which all showed comparable between the ocrelizumab/methotrexate and placebo/methotrexate groups, ranging from 1 to 4.7% (52, 58). Of the patients that developed an ADA response, there was no correlation between immunogenicity and reduced efficacy or adverse effects of the drug (52).

For other new-generation anti-CD20 mAb, studies showed development of ADA responses but no effect on the efficacy of the drugs (111). This shows that the new generation of anti-CD20 mAbs is as efficient as the ‘old’ generation, but with a better safety profile in terms of immunogenicity (52).

#### CD38-inhibiting mAbs

CD38 functions as cell surface ectoenzyme. It contributes to the modification of NAD^+^ released from damaged cells in inflammation, to immunosuppressive extracellular adenosine. CD38 might also play a role in the promotion of tumour growth *via* the suppression of effector T-cell responses in a tumour environment (112). An anti-CD38 specific mAb used in the clinic is daratumumab, which shows to be effective in multiple myeloma (112).

##### Immunogenicity of Daratumumab

Daratumumab is generated from CD38-immunized transgenic mice carrying the genomic loci encoding human IgH and IgI (112). When administered intravenously, the drug shows a low risk of immunogenicity (1.3% of the patients). The presence of antibodies increased with increased dosage. The antibodies that were formed, were neutralizing but did not affect pharmacokinetics (59, 60).

#### CD52-inhibiting mAbs

The membrane glycoprotein CD52 is expressed by lymphocytes, monocytes, subsets of dendritic cells and subsets of epithelial cells. Inhibiting this glycoprotein causes the depletion of lymphocytes and blood dendritic cells *in vivo*, making it a target for mAbs designed to treat graft versus host disease in bone marrow transplantation, relapsing multiple sclerosis and chronic lymphocytic leukaemia (113). The anti-CD52 mAb used in the clinic is alemtuzumab, which is a humanized mAb.

##### Alemtuzumab

Alemtuzumab is the first humanized mAb. The humanization was performed by removing rodent constant regions and grafting the complementarity-determining regions onto human framework regions. This process, however, seemed not enough to prevent ADA responses to occur. Studies reveal the presence of antibodies in 85% of cases within 24 months, even though these have no reported clinical significance (60, 61). It was mentioned that the development of these ADAs can become more problematic with increasing number of treatment cycles and will therefore need monitoring in the clinic, as they can persist for years (61).

#### HER2-inhibiting mAbs

The human epidermal growth factor receptor-2 (HER2) is a proto-oncogene, encoding a transmembrane tyrosine kinase receptor (114). Amplification of the HER2-gene and overexpression of this tyrosine kinase receptor is observed in 20-30% of women with breast cancer, which is associated with a worse prognosis. Amplification or overexpression of HER2 is believed to have a role in the pathogenesis of breast cancer (115). Trastuzumab and pertuzumab are anti-HER2 mAbs currently used in the clinic. The mAbs are used separately, but also in combination (62, 63, 65).

##### Immunogenicity of Trastuzumab

Trastuzumab, a humanized anti-HER2 mAb, inhibits the growth of HER2-overexpressing tumour cells. It provides a significant survival benefit on its own and in combination with chemotherapeutic agents (115). Studies show the relation between the number of administered doses and the percentage of ADA-positive patients up to 3 administered doses. Higher administered doses present a dose-dependent reduction in percentage of positive samples which could be due to a developed tolerance after initial administered doses. Only a low percentage (1%) of the patients show ADA responses, but these do have high titers of anti-trastuzumab antibodies. These ADAs do not impact the efficacy of the drug (62–64).

##### Immunogenicity of Pertuzumab

Pertuzumab blocks the formation of the HER2-dimer and its receptor, by binding to HER2. It thereby inhibits the activation of the Pi3K-PKB/Akt signalling pathway Thereby, pertuzumab inhibits cancer cell activation, slows tumour growth and reduces the recurrence rate (65). However, a study shows that 30% of patients included in the trial develop an ADA response, 70 days after administration. The antibodies did not neutralize or affect pertuzumab (65).

#### EGFR-inhibiting mAbs

The epidermal growth factor receptor (EGFR) is a tyrosine kinase receptor that plays a role in the homeostatic regulation of normal cells and carcinogenesis of epithelial malignancies (116). There are currently four anti-EGFR mAbs approved for use in the clinic; cetuximab, panitumumab, nimotuzumab and necitumumab.

##### Immunogenicity of Cetuximab

Cetuximab is a human/mouse chimeric IgG1 mAb that blocks the interaction between EGFR and its ligands, by which the downstream RAS-signalling pathway and ERK-activation is inhibited (117). In studies assessing the immunogenicity, only non-neutralizing anti-cetuximab antibodies were found in 5% of the patients, indicating that there is no relation between these antibodies and the pharmacokinetics or efficacy and safety profile (66–69).

##### Immunogenicity of Panitumumab

Panitumumab is a fully human IgG2 antibody that, like cetuximab, binds the EGFR (117). Immunogenicity of panitumumab in combination therapy with cytostatics is similar to the immunogenicity observed in panitumumab monotherapy (1.8%). The antibodies formed do not have a clinical effect as these do not alter the pharmacokinetics or safety profile (70–72).

##### Immunogenicity of Nimotuzumab

Nimotuzumab is a humanized IgG1-mAb that binds the EGFR to inhibit the EGFR-pathway (73, 116). In studies testing the immunogenicity, no anti-nimotuzumab antibodies were detected (73).

##### Immunogenicity of Necitumumab

Necitumumab is a fully human IgG2 anti-EGFR antibody that stimulates natural killer (NK) cell-driven cytotoxicity against tumour cells *via* the interaction of its constant region and CD16 receptor on NK-cells (118). Clinical trials show neutralizing anti-necitumumab antibodies in 1.4% of the tested population (74).

#### Immune Checkpoint Inhibitor-Inhibiting mAbs

The cytotoxic T-lymphocyte-associated antigen 4 (CTLA-4) and programmed death 1 (PD-1) immune checkpoint pathways play key roles in the peripheral tolerance. CTLA-4 is often considered ‘leader’ of immune checkpoint inhibitors, due to its ability to prevent autoreactive T-cells at initial stage of naïve T-cell activation, primarily in lymph nodes. The previously activated T-cells are in a later stage of the immune response regulated by PD-1 (119–121). In cancer immunotherapy, tumour cells have developed ways to avoid recognition by T-cells, by which the cells can take advantage of peripheral tolerance (120).

##### Immunogenicity of Ipilimumab

Ipilimumab is a fully human IgG1 mAb. Neutralizing anti-ipilimumab antibodies have been found in 26% of the patients after administration of the drug. These neutralize drug target binding or enhance drug clearance, which both cause loss of treatment efficacy and suboptimal levels of active drugs. Also, several hypersensitivity reactions have been registered in relation to the ADA response. Studies even show shortened overall survival and shortened progression-free survival (75).

##### Immunogenicity of Pembrolizumab

Pembrolizumab is a humanized IgG4 mAb, targeting immune checkpoint PD-1. The incidence of ADA responses against pembrolizumab is low, as anti-pembrolizumab antibodies were found in 3.4% of the patients. The antibodies are non-neutralizing; this immunogenicity has no clinically relevant effects on exposure, safety or efficacy of the drug (76).

##### Immunogenicity of Nivolumab

Nivolumab is a fully human IgG4 mAb that targets the immune checkpoint PD-1. The incidence of ADA responses against nivolumab is low (12.7% of patients were ADA-positive), and the antibodies that are formed do not affect the pharmacokinetic profile or efficacy of the drug (73, 77, 78).

##### Immunogenicity of Atezolizumab

Atezolizumab is a humanized IgG1 mAb that binds programmed death-ligand 1 (PD-L1), thereby inhibiting interactions between PD-1 and CD80 receptors. The drug shows high variability of ADA response rates, ranging from 13 to 54%. The antibodies formed, however, did not show impact on the pharmacokinetic profile and efficacy of the drug (79).

##### Immunogenicity of Avelumab

Avelumab is a fully human IgG1 mAb which targets PD-L1. An ADA response was observed in 14 to 19% of the tested patients after 2.5 months. The presence of the antibodies, however, had no influence on pharmacokinetics or safety of the drug (80, 81).

### Interleukin-targeted mAbs

#### Immunogenicity of Tocilizumab

Tocilizumab is a humanized IgG1 mAb that blocks the binding between IL-6 and IL-6R, by which it inhibits IL-6 activity. Studies show low immunogenicity of the drug (<2% of patients), with the antibodies present not affecting pharmacokinetics or safety profiles (82, 122).

#### Immunogenicity of Ustekinumab

Ustekinumab is a human IgG1 mAb that targets the shared p40 subunit of IL-12 and IL-23. ADA responses occur in 5% of patients, of which the majority consist of neutralizing antibodies which affect the effectivity of the drug (83, 84).

#### Immunogenicity of Secukinumab

Secukinumab is a fully human IgG1 mAb that targets IL-17. ADA responses were seen in <1% of patients, and was not linked to immunogenicity-related adverse effects and therefore there is no clinical effect on the efficacy and/or the pharmacokinetics of the drug (85).

#### Immunogenicity of Ixekizumab

Ixekizumab is a humanized mAb targeted against IL-17A. In clinical trials, 17% of patients receiving the drug developed non-*nt*ADAs where the presence of the anti-ixekizumab antibodies did not interfere with serum concentrations and drug efficacy (86, 87).

### IgE-inhibiting mAbs

IgE antibodies are known for their effector functions and their function in mediating allergic reactions. This effector function is an interesting target for mAb (123).

#### Immunogenicity of Omalizumab

Omalizumab is an anti-IgE mAb which inhibits the effector function of IgE, as well as the activation of mast cells and basophils (124). No antibodies against omalizumab are detected (88).

### VLA4-inhibiting mAbs

Very late active antigen (VLA)-4 functions as receptor for the vascular cellular adhesion molecule 1 (VCAM-1) (93).

#### Immunogenicity of Natalizumab

Natalizumab is a IgG4 mAb targeting VLA-4. The drug is prescribed in multiple sclerosis-patients. Here, it effectively reduces the disease activity and decreases the risk of disability progression (94). In 4 to 14% of the patients, anti-natalizumab antibodies are found. The presence of these antibodies is associated with an increased risk for infusion-related adverse effects, but it also causes a reduced clinical effect (93–95).

## HLA Haplotypes as biomarker to Predict the Occurrence of ADA Responses in Vulnerable Individuals

Our review indicates that there is no clear path to say if and which ADA responses will occur based on the structure of the mAb. The summarising **Table 1** clearly shows an overview of the ADA responses against commonly used mAbs in the clinic. The range of frequencies of ADAs reported are due to various factors, few of them being the lack of standardized tests to detect ADAs, different patient populations used in the studies, different diseases present in the studied populations and so on. This is especially true for the older molecules like infliximab, adalimumab and rituximab, but is also an important note for the ‘newer’ molecules.

Recently, studies have focused on the possibility to predict the occurrence of ADA responses in individual patients. There is evidence that patients carrying specific HLA haplotypes are more vulnerable to form ADAs and this may be useful to personalize mAb treatment (125–129).

The human leukocyte antigen (HLA) system is involved in rejecting foreign entities that enter the body. They encode highly polymorphic cell surface molecules (130). Due to their role in the immune response against foreign entities, HLA haplotypes are believed to play an important role in the formation of ADA responses. For example, the relation between anti-infliximab antibodies and the HLA-DRB1 alleles has been studied, as well as the relation between anti-adalimumab antibodies and HLA-DRB1*03 (128).

Another study mentions the link between genetic variations in HLA and a higher risk of anti-TNFα antagonist antibodies. A direct causal link has not been found, but the results suggest that polymorphisms in HLA-DQA1*05 not only cause immunogenicity against the drug, but also a lower efficacy and affected pharmacokinetic profile. A single nucleotide variation (rs2097432) that shows increased risk of immunogenicity against adalimumab and infliximab have been described (131–133). In a study covering this variation, the allele frequency of rs2097432 was 25.5%. The risk of developing ADA responses was higher in the variant carriers (adjusted HR = 7.29). The carriers of this variant also had an increased risk of loss of response (adjusted HR = 2.34) and discontinuation (adjusted HR = 2.27) (132).

The possibility for these HLA-haplotypes to predict the occurrence of ADA responses was also tested for rituximab and atezolizumab (134, 135). Here, the associated alleles were HLA-DRB1*01:01 for all ADA (p = 3.4*10^−5^, odds ratio = 1.96), and HLA-DQA1*01:01 when considering ntADA (p = 2.8 x 10^−7^, odds ratio = 2.31). These alleles both occur in common HLA haplotypes. The study indicates that the HLA class II genotype plays a role in the development of anti-atezolizumab antibodies. However, the study also suggests that these genetic factors are yet insufficient as clinically meaningful predictors of ADA responses (135).

The explorative studies on the relation of the HLA haplotypes and variants need validation before they can be used as reliable biomarker in the clinic. This is, however, by far not enough information to base all ADA responses against all mAbs upon, as it is unlikely that there is one general biomarker to predict ADA formation for all different mAbs. Therefore, future research should be focused on the possibility to use these variants in HLA haplotypes as biomarker in the clinic, to prevent the immunogenicity against the mAb and thereby increase the benefit patients can experience from the drugs.

## Future Perspectives on the Use of HLA Haplotypes to Predict Vulnerable Patients

In conclusion, the occurrence of antidrug antibody responses against mAb is a serious clinical concern. However, despite many mAbs displaying these ADA responses, it is inappropriate to directly compare the incidence rates of these responses across molecules. The immunogenicity incidence rates are highly dependend on the type of assay that is used to identify the ADA response, the dosing regimen that is prescribed, the time points on which the samples for the ADA-assays are taken, the route of administration of the mAb, the state of the underlying disease, the use of concurrent immunosuppressive treatment and many other factors. Therefore, the direct comparison of immunogenicity rates across the biologicals described in this paper is difficult.

Additionally, it is currently not possible to predict the occurrence of ADA responses, neither based upon mAb related factors nor on patient related factors. While not being the only plausible risk factor for ADA formation, studies that explore the possibility to predict ADA formation suggest the involvement of genetic variants in HLA haplotypes. For some drugs and in some diseases, the possibility to use these genetic variants as biomarkers has been studied, but for the majority of mAbs this is yet unexplored.

In the future, HLA haplotype identification might play an important role in the prevention of ADA responses, thereby not only improving the experience of the patients receiving drugs, but also minimizing hospitalization due to ADA responses and improving the treatment of patients as the correct drugs that do not elicit an ADA response can be prescribed quicker. This technique can not only be applied to MoAbs, but can also be explored to be helpful in the prediction of reactions of the immune system against other classes of drugs.

## Author Contributions

RM wrote the review under supervision of H-JG. All authors contributed to the article and approved the submitted version.

## Conflict of Interest

The authors declare that the research was conducted in the absence of any commercial or financial relationships that could be construed as a potential conflict of interest.

## Publisher’s Note

All claims expressed in this article are solely those of the authors and do not necessarily represent those of their affiliated organizations, or those of the publisher, the editors and the reviewers. Any product that may be evaluated in this article, or claim that may be made by its manufacturer, is not guaranteed or endorsed by the publisher.
